# Effectiveness of inpatient and outpatient strategies in increasing referral and utilization of cardiac rehabilitation: a prospective, multi-site study

**DOI:** 10.1186/1748-5908-7-120

**Published:** 2012-12-13

**Authors:** Sherry L Grace, Kelly L Angevaare, Robert D Reid, Paul Oh, Sonia Anand, Milan Gupta, Stephanie Brister, Donna E Stewart

**Affiliations:** 1 School of Kinesiology and Health Science, York University, 4700 Keele St, Toronto, Canada; 2 Toronto General Hospital, University Health Network, 200 Elizabeth St, Toronto, Canada; 3 Faculty of Medicine, University of Toronto, 1 King’s College Circle, Toronto, Canada; 4 Mackenzie Health, 10 Trench Street, Richmond Hill, Canada; 5 Cardiac Program, Toronto Rehabilitation Institute, University Health Network, 347 Rumsey Road, Toronto, Canada; 6 Minto Prevention and Rehabilitation Centre, University of Ottawa Heart Institute, 40 Ruskin Street, Ottawa, Canada; 7 Clinical Epidemiology and Biostatistics, McMaster University, 237 Barton St. E, Hamilton, Canada; 8 William Osler Health Centre, 2100 Bovaird Drive East, Brampton, Canada

**Keywords:** Cardiac rehabilitation, Patient care management, Cardiovascular diseases

## Abstract

**Background:**

Despite the evidence of benefit, cardiac rehabilitation (CR) remains highly underutilized. The present study examined the effect of two inpatient and one outpatient strategy on CR utilization: allied healthcare provider completion of referral (a policy that had been endorsed and approved by the cardiac program leadership in advance; PRE-APPROVED); CR intake appointment booked before hospital discharge (PRE-BOOKED); and early outpatient education provided at the CR program shortly after inpatient discharge (EARLY ED).

In this prospective observational study, 2,635 stable cardiac inpatients from 11 Ontario hospitals completed a sociodemographic survey, and clinical data were extracted from charts. One year later, participants were a mailed survey that assessed CR use. Participating inpatient units and CR programs to which patients were referred were coded to reflect whether each of the strategies was used (yes/no). The effect of each strategy on participants’ CR referral and enrollment was examined using generalized estimating equations.

**Results:**

A total of 1,809 participants completed the post-test survey. Adjusted analyses revealed that the implementation of one of the inpatient strategies was significantly related to greater referral and enrollment (PRE-APPROVED: OR = 1.96, 95%CI = 1.26 to 3.05, and OR = 2.91, 95%CI = 2.20 to 3.85, respectively). EARLY ED also resulted in significantly greater enrollment (OR = 4.85, 95%CI = 2.96 to 7.95).

**Conclusions:**

These readily-implementable strategies could significantly increase access to and enrollment in CR for the cardiac population. The impact of these strategies on wait times warrants exploration.

## Background

Cardiovascular disease (CVD) remains the leading cause of mortality worldwide [1], and is mainly attributable to modifiable risk factors, such as hypertension, dyslipidemia, obesity, smoking, and a sedentary lifestyle [2]. Cardiac rehabilitation (CR) offers a comprehensive approach to chronic disease management by addressing these risk factors. CR programs serve to maintain and enhance cardiovascular health through individualized programs of exercise, secondary prevention, education, and counseling, offered in supervised or home-based settings. CR has been shown to significantly reduce morbidity and mortality by 25% when compared to usual care [3,4], a similar degree to widely accepted medication regimens, such as statins, aspirin, and beta-blockers [3,5]. Based on this evidence, CR is recommended as the standard of care in clinical practice guidelines for acute coronary syndrome and revascularization [6,7] and other cardiac populations [6,8,9]. Performance measures have also been developed [10].

Unfortunately, utilization of CR remains suboptimal, as evidenced by data from the United States (US), Canada, and the United Kingdom (UK) demonstrating that only 20% to 30% of eligible cardiac patients receive CR after hospital discharge [10-12]. Reasons for low CR enrollment are multi-factorial, evident at the patient, health professional, and healthcare system level [13,14]. In an effort to overcome these barriers, a variety of strategies have been developed and show promise in increasing CR enrollment [7]. These include ‘systematic’ CR referral, providing patients with theory-based motivational letters or pamphlets, and discussions endorsing CR at the bedside [15-18]. Previous work from our group has demonstrated the positive impact of standard order sets that include CR, eReferral, and discussion at the bedside on enrollment rates [18].

Key opinion leaders [19] have identified two other strategies used by inpatient units and one by CR programs that are hypothesized to improve CR referral and utilization: cardiac program leadership endorsement of a policy for referral by an allied health professional of all indicated patients (PRE-APPROVED); CR intake appointment booked prior to hospital discharge (PRE-BOOKED); and early outpatient education provided at the CR program shortly after inpatient discharge (EARLY ED). Through secondary analysis of a previously-published cohort study [18], the objective of the present study was to examine the effects of these untested strategies on CR referral and utilization for the first time.

## Methods

### Design and procedure

This study presents secondary analyses of a published cohort study designed to assess other inpatient-targeted referral strategies [18,19]. The design of this multi-centre study was prospective and quasi-experimental, assessing comparative effectiveness of each of the following three strategies in increasing CR utilization compared to patients receiving care without the strategies: PRE-APPROVED, PRE-BOOKED, and EARLY ED. Each strategy was tested individually in comparison to patients who were not exposed to that specific strategy, because they were not mutually exclusive. With regard to the former, given that clinical practice guidelines promote CR referral as the standard of care, some cardiac wards have standing orders in place so that nurses, allied healthcare professionals, and ward clerks can facilitate referral form completion and submission for indicated patients as pre-approved by the cardiac program leadership. The forms would be specific to the CR program to which patients are referred. There is no requirement for patients for this process to occur, however it is assumed that verbal consent is secured. This process is perceived to overcome referral failure because there is no time demand for physicians. With regard to the second strategy, inpatients are provided with a CR intake appointment prior to discharge. This would be done routinely for all patients providing verbal consent. Finally, with regard to the third outpatient strategy, here CR programs arranged interprofessional education sessions for outpatients shortly after referral, but before commencing the CR program. These patient education sessions generally conveyed information regarding cardiac risk factors and their reduction, cardiac medications, the nature of the CR program, and answering any questions patients may have. While this is not a referral strategy *per se*, more patients may ultimately enroll in CR if they learned about the CR program at a time when they are more motivated from their recent cardiac episode and discharge.

All participating institutions’ research ethics boards reviewed and approved the study. Medically stable cardiac inpatients from 11 community and academic hospitals bordered by Windsor, Sudbury, and Ottawa, Ontario were approached to participate between 2006 and 2008. CR services are covered through provincial health insurance in these jurisdictions. CR programming is guided by the Canadian Association of Cardiac Rehabilitation guidelines [20], averaging five months in duration, with a median of two supervised sessions per week [21]. Overall there were 17 cardiac wards at these hospitals, as one hospital had three cardiac wards, and four hospitals had two cardiac wards each (*i.e.*, surgery vs other). Of these 17 wards, 11 (64.7%) used the PRE-APPROVED strategy, and four (23.5%) used the PRE-BOOKED strategy.

Upon providing informed consent, eligible patients completed a sociodemographic survey. Clinical data were extracted from medical charts. Participants were then mailed a follow-up survey one year later, assessing self-reported CR referral, site of referral, and utilization. The 61 CR programs to which 1,156 (64.9%) patients reported referral were contacted. Some of these programs were academic and others non-academic, sited in hospital and community settings, and supervised and home-based program models were offered. Overall, six (9.8%) CR programs used early outpatient education.

### Participants

A total of 2,635 stable cardiac inpatients were recruited from these 17 wards. Inclusion criteria were: confirmed acute coronary syndrome diagnosis, patients who had undergone percutaneous coronary intervention (PCI) or coronary artery bypass graft surgery (CABG), patients with a concomitant diagnosis of heart failure, eligibility for CR based on guidelines of the Canadian Association of Cardiac Rehabilitation [20], and proficiency in English, French, or Punjabi (surveys were translated into each of these languages). Diagnosis of acute coronary syndrome was confirmed through patient chart review of detailed history, focused physical examination, diagnostic ECG changes, and/or troponin levels above the 99^th^ percentile of normal. Patients were excluded if they had participated in CR within the past two years, or had a significant orthopedic, neuromuscular, visual, cognitive, or non-dysphoric psychiatric condition that precluded CR participation.

### Measures

#### Sociodemographic and clinical characteristics

Self-reported sociodemographic characteristics assessed in the survey through forced-choice response options included marital status, education level, ethnocultural background, family income, and work status. In addition, patients were coded as ‘rural living’ if they lived beyond a 30-minute drive time of the hospital [22]. Sociodemographic data obtained from the medical chart included date of birth and gender.

With regard to clinical characteristics, the patient survey included the Duke Activity Status Index [23] to assess functional status. Nature of cardiac condition or procedure, presence of CVD risk factors (*e.g.*, family history, dyslipidemia, diabetes, hypertension, obesity), and comorbidities were also obtained from the medical chart.

#### Independent variable: CR referral strategy

Meetings with the clinical staff from all inpatient units and study investigators were held to understand the processes of CR referral, resulting in categorization of two inpatient referral strategies: (1) PRE-APPROVED and (2) PRE-BOOKED. Participants reported to which CR program they were referred. These programs were contacted to verify whether they offered (3) EARLY ED.

Participating inpatient units and CR programs to which patients reported referral were all coded to reflect whether the applicable strategy was used, dichotomized as ‘yes’ or ‘no.’ Each patient was then coded as ‘exposed’ or ‘not exposed’ to each of the three strategies. Because the strategies were not mutually exclusive, three pragmatic tests were undertaken, one for each strategy compared to those patients not exposed to that specific strategy.

#### Dependent variables: Cardiac rehabilitation referral and utilization

Participants self-reported whether or not they were referred to cardiac rehabilitation (yes/no). The rate of referral following each of the two inpatient strategies was compared to those not referred by the particular strategy.

Utilization was operationalized as whether they attended a CR intake assessment (yes/no; *i.e.*, enrollment) and the degree of participation (*i.e.*, self-reported percentage of prescribed sessions attended). The rate of enrollment following each of the three strategies was compared to those not referred by the particular strategy.

#### Statistical analyses

Sociodemographic and clinical characteristics of participants exposed to each CR utilization strategy (by ward or CR program) were compared to those not exposed to the strategy using chi-square analyses for categorical variables and t-test for continuous variables.

A descriptive examination of CR referral (for inpatient strategies only) and utilization rates by each strategy was performed. Two generalized estimating equations were then computed to take into consideration the nested nature of patients within hospitals, to test for differences in CR referral and enrollment by strategies. The models were adjusted for sociodemographic and clinical differences with a p value <0.05 identified through bivariate testing outlined above. A p value of <0.05 was considered statistically significant for all tests. SPSS statistical software (version 18.0; SPSS Inc, Chicago, Illinois) was used for all analyses.

## Results

### Respondent characteristics

Of the 5,767 inpatients approached, 1,449 were ineligible and 2,635 of the remaining 4,318 consented to participate (61.0% response rate). At one-year post-recruitment, 1,809 (68.7% retained) completed the follow-up survey and 826 were lost to follow-up. The reasons for loss and characteristics of participants retained versus those not retained are presented elsewhere [18]. Retained participants were more likely to be married, have undergone CABG, and less likely to smoke, or have diabetes than non-retained participants. Patients who declined to participate were more likely to be younger, working, to be of non-white race, a current smoker, have diabetes, and less likely to be married and have undergone CABG or valve surgery than retained participants.

**Figure 1:**
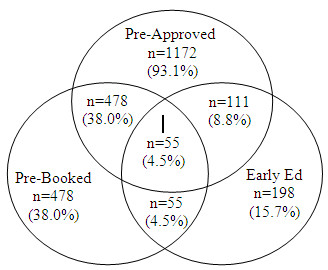
Venn diagram depicting patient exposure to the three strategies, N = 1,259.

Table 1 displays the number of wards adopting each of the two inpatient strategies. Table 2 displays the number of CR sites offering early outpatient education. Also shown is the number of participants exposed to these strategies. This interaction between the interventions is depicted in Figure 1, and controlled for through multivariate generalized estimating equations (GEE).

**Table 1:** Sociodemographic and clinical characteristics of participants by inpatient CR referral strategy

**Characteristic**	**PRE-APPROVED**		**PRE-BOOKED**		**TOTAL**
	**Yes**	**No**	**Yes**	**No**	
	**(11 wards)**	**(6 wards)**	**(4 wards)**	**(13 wards)**	**(17 wards)**
	**n = 1172**	**n = 637**	**n = 478**	**n = 1331**	**n = 1809**
**Sociodemographic**					
Mean age, yrs (SD)	65.53 (10.20)	65.14 (10.75)	64.91 (9.93)	65.56 (10.55)	65.39 (10.40)
Gender, female, n (%)	256 (21.8)	196 (30.8)^***^	91 (19.0)	361 (27.1)^‡^	452 (25.0)
White ethnocultural background, n (%)	968 (86.4)	478 (78.0)^***^	399 (88.7)	1047 (81.6)^**^	1446 (83.4)
Married, n (%)	923 (79.0)	469 (75.4)	376 (79.0)	1016 (77.3)	1392 (77.8)
Some post-secondary education or greater, n (%)	851 (74.1)	461 (76.3)	345 (72.9)	967 (75.5)	1312 (74.8)
Retired, n (%)	592 (52.4)	313 (51.2)	226 (49.3)	679 (53.0)	905 (52.0)
Family income ≥$50,000 CAD (approx. $28,500 USD), n (%)	473 (50.8)	257 (48.7)	207 (55.9)	523 (48.0)^**^	730 (50.0)
Rural living, n (%)	196 (16.7)	117 (18.4)	69 (14.4)	244 (18.3)	313 (17.3)
**Clinical**					
Cardiac condition/procedure, n (%)					
MI	366 (31.4)	136 (21.6)^***^	136 (28.6)	366 (27.7)	502 (28.0)
PCI	301 (25.7)	301 (47.9)^***^	27 (5.7)	575 (43.5)^***^	602 (33.5)
CABG	550 (47.0)	193 (30.7)^***^	363 (76.3)	380 (28.7)^***^	743 (41.3)
Heart failure	135 (11.5)	59 (9.4)	51 (10.7)	143 (10.8)	194 (10.8)
Arrhythmia	151 (12.9)	72 (11.4)	76 (16.0)	147 (11.1)^**^	223 (12.4)
Valve repair/replacement	118 (10.1)	35 (5.6)^**^	62 (13.0)	91 (6.9)^***^	153 (8.5)
Diabetes, n (%)	330 (29.5)	187 (35.6)^*^	139 (30.5)	378 (31.8)	517 (31.5)
Mean BMI (SD)	29.31 (5.66)	28.54 (5.01)^*^	29.23 (4.79)	28.95 (5.71)	29.04 (5.45)
Family history of CVD, n (%)	596 (63.3)	258 (68.4)	262 (62.7)	592 (65.7)	854 (64.7)
Hypertension, n (%)	798 (72.0)	441 (78.3)^**^	339 (73.7)	900 (74.3)	1239 (74.1)
Dyslipidemia, n (%)	823 (80.3)	461 (84.9)^*^	368 (82.7)	916 (81.6)	1284 (81.9)
Smoker, n (%)	66 (5.8)	45 (7.4)	25 (5.4)	86 (6.7)	111 (6.4)
Mean DASI score (SD)	26.97 (17.07)	29.65 (17.26)^**^	22.73 (16.28)	29.76 (17.12)^***^	27.91 (17.18)
Comorbidities present, n (%)	709 (66.0)	405 (71.4)^*^	304 (67.9)	810 (67.8)	1114 (67.8)

*Note.* n = 1809. PRE-APPROVED = physician signature not required (CR referral pathway endorsed and pre-approved by cardiac program leadership), PRE-BOOKED = CR intake appointment booked pre-hospital discharge.

^*^p < 0.05; ^**^p < 0.01; ^***^p < 0.001.

BMI = body mass index; CABG = coronary artery bypass grafting; CR = cardiac rehabilitation, CVD = cardiovascular disease; DASI = Duke Activity Status Index; MI = myocardial infarction; PCI = percutaneous coronary intervention; SD = standard deviation.

**Table 2:** Sociodemographic and clinical characteristics of participants by early outpatient education at CR

**Characteristic**	**EARLY ED**	
	**Yes**	**No**
	**(n = 6 CR programs)**	**(n = 55 CR programs)**
	**(n = 198)**	**(n = 1611)**
**Sociodemographic**		
Mean age, yrs (SD)	65.28 (10.39)	65.40 (10.40)
Gender, female, n (%)	40 (20.2)	412 (25.6)
White ethnocultural background, n (%)	148 (74.7)	1298 (84.6)^***^
Married, n (%)	156 (79.2)	1236 (77.6)
Some post-secondary education or greater, n (%)	164 (84.5)	1148 (73.6)^**^
Retired, n (%)	98 (49.7)	807 (52.3)
Family income ≥$50,000 CAD (approx. $28,500 USD), n (%)	108 (61.4)	622 (48.5)^**^
Rural living, n (%)	26 (13.1)	287 (17.8)
**Clinical**		
Cardiac condition/procedure, n (%)		
MI	45 (22.7)	457 (28.6)
PCI	47 (23.7)	555 (34.7)^**^
CABG	103 (52.0)	640 (40.0)^**^
Heart failure	18 (9.1)	176 (11.0)
Arrhythmia	15 (7.6)	208 (13.0)^*^
Valve repair/replacement	24 (12.1)	129 (8.1)
Diabetes, n (%)	49 (28.2)	468 (31.9)
Mean BMI (SD)	28.47 (4.68)	29.10 (5.52)
Family history of CVD, n (%)	88 (67.2)	766 (64.5)
Hypertension, n (%)	134 (76.1)	1105 (73.9)
Dyslipidemia, n (%)	149 (85.1)	1135 (81.5)
Smoker, n (%)	8 (4.1)	103 (6.7)
Mean DASI score (SD)	26.50 (16.29)	28.08 (17.28)
Comorbidities present, n (%)	121 (69.1)	993 (67.7)

*Note.* n = 1809. EARLY ED = early outpatient education.

^*^p < 0.05; ^**^p < 0.01; ^***^p < 0.001.

BMI = body mass index; CABG = coronary artery bypass grafting; CAD = Canadian dollars’ CR = cardiac rehabilitation, CVD = cardiovascular disease; DASI = Duke Activity Status Index; MI = myocardial infarction; PCI = percutaneous coronary intervention; SD = standard deviation; USD = United States dollars.

There were 637 (35.2%) participants who were not exposed to either inpatient strategy, and 550 (30.4%) were not exposed to any inpatient or outpatient CR utilization strategy. When comparing the characteristics of participants exposed to any CR strategy versus none, there were no significant differences by age (p = 0.93), but men (72.3%) were significantly more likely to be exposed to a strategy then women (61.9%; p<0.001). Two hundred and ninety-eight (47.5%) and 211 (39.1%) of participants not exposed to any utilization strategy were referred to CR, respectively. Two hundred and forty-three (39.8%) and 178 (33.8%) participants not exposed enrolled, respectively.

In-hospital sociodemographic and clinical characteristics are presented by strategy in Tables 1 and 2. As shown, due to lack of randomization, there were significant differences in these characteristics by strategy, and were therefore controlled for in subsequent analyses.

### Effect of referral strategies

Table 3 compares CR referral and enrollment rates by referral strategy. Referral and enrollment rates ranged from 74% to 81% and 65% to 84%, respectively, for sites that used a referral strategy compared with sites that did not (48% to 59%, 40% to 53%, respectively).

**Table 3:** Cardiac rehabilitation referral and utilization rates by strategy

**Strategy**	**Referred, n (%)**	**Enrolled, n (%)**	**Percent of prescribed CR sessions attended of those referred, mean (SD)**
PRE-APPROVED			
Yes	858 (74.3)	735 (65.2)	82.73 (27.04)
No	298 (47.5)	243 (39.8)	84.28 (26.52)
PRE-BOOKED			
Yes	382 (81.1)	324 (70.3)	80.64 (28.31)
No	774 (59.0)	654 (51.3)	84.37 (26.09)
EARLY ED			
Yes	-	159 (84.1)	80.43 (27.53)
No	-	819 (52.9)	83.63 (26.78)

Note. Caution is warranted interpreting this table due to interaction between the interventions.

Referred participants were sent to 1 of 52 CR programs (includes private and community-operated). As shown in Table 2, there were six CR programs that implemented early outpatient education. Also shown in Table 3 is the self-reported percentage of prescribed sessions attended, which was not shown to differ by strategy. In other words, once patients did enroll in a program, they attended a similar proportion of pre-scheduled visits.

Table 4 displays the results of the two generalized estimating equations, with odds ratios for CR referral (inpatient strategies only) and enrollment by strategies. Using generalized estimating equations to control for hospital site, as well as sociodemographic and clinical characteristics shown to differ significantly in Tables 1 and 2, results showed that implementation of the PRE-APPROVED inpatient strategy was related to significantly greater referral. Other variables that contributed significantly to referral were white ethnocultural background (OR = 0.55, 95%CI = 0.33 to 0.91), previous myocardial infarction (OR = 1.79, 95%CI 1.14 to 2.82), previous coronary artery bypass grafting (OR = 2.79, 95% CI = 1.62 to 4.79), having diabetes (OR = 0.64, 95% CI = 0.44 to 0.94), and activity status (OR = 0.99, 95% CI = 0.98 to 0.99). In the case of CR enrollment, PRE-APPROVED and the outpatient strategy EARLY ED both resulted in significantly greater enrollment. Other variables that contributed significantly to enrollment were greater education (OR = 1.50, 95% CI = 1.12 to 2.00), greater family income (OR = 1.96, 95% CI = 1.53 to 2.51), and previous coronary artery bypass grafting (OR = 1.39, 95% CI = 1.77 to 3.24).

**Table 4:** GEE analysis of cardiac rehabilitation referral and enrollment rates by strategy

**Variable**	**Wald statistic**	**Adjusted OR**	**95% CI**
CR referral^a^			
PRE-APPROVED	8.90^*^	1.96	1.26-3.05
PRE-BOOKED	1.79	1.49	0.83-2.69
CR enrollment^b^			
PRE-APPROVED	56.54^**^	2.91	2.20-3.85
PRE-BOOKED	.01	1.00	0.71-1.42
EARLY ED	39.16^**^	4.85	2.96-7.95

*Note.* Reference used = patient not exposed to specific strategy.

CI = confidence interval; OR = odds ratio; MI = myocardial infarction; PCI = percutaneous coronary intervention; CABG = coronary artery bypass grafting; BMI = body mass index; DASI = Duke activity status index.

^a^Adjusted for hospital site, sex, ethnocultural background, MI, PCI, CABG, valve repair/replacement, diabetes, dyslipidemia, BMI, DASI score, hypertension, comorbidities present.

^b^Adjusted for hospital site, ethnocultural background, education, income, PCI, CABG, arrhythmia.

^*^p < 0.01; ^**^p < 0.001.

## Discussion

Despite its known benefits, only approximately 30% of eligible inpatients subsequently utilize outpatient CR [11,13]. Accordingly, the American Heart Association recently issued both a Science and Presidential Advisory on the importance of expanding access to CR and the valuable role of healthcare professionals in increasing referral to CR [7,24]. Fittingly, through interviews conducted with clinical staff, the present study identified and subsequently evaluated the effect of three untested strategies used by inpatient units and CR programs to improve CR referral and utilization. The inpatient strategy of pre-approved referral significantly increased referral and enrollment in these evidence-based programs. Provision of early outpatient education at the CR program was also related to significantly greater enrollment. Ultimately, the strategies resulted in rates of use around 65% to 84%, which was approximately two to five times greater than usual practice. The rates of CR referral and enrollment observed in this study are encouraging and come close to the Canadian national published targets of 85% referral and 70% enrollment [6]. The positive effect of these strategies on referral and enrollment rates are concordant with studies published examining the effect of innovative referral strategies [15-18]. Degree of CR participation was uniformly high after enrollment, which suggests that ‘if we refer, they will come.’ It is incumbent on the healthcare community to adopt these strategies to ensure universal access to this evidence-based care.

Buy-in of standard CR referrals by healthcare providers and administrators likely has the advantage of making the referral process habitual and ensuring all members of the patient care team are supporting the referral process. Some physicians may be reticent to have their patients uniformly referred, as indeed it has been shown that some physicians have less than positive perceptions of the benefits of CR [25,26]. Ultimately though, the referral is made to education, as exercise is not initiated until another careful medical assessment occurs at the CR site. Moreover, a patient’s health status will generally change from the time of discharge to the time of CR intake, so degree of readiness for exercise at the time of discharge should not necessarily negate referral. CR programs undertake extensive medical assessments at program initiation to ensure patients are indeed suitable for the program and to tailor services to their health status at that time. In addition, there is a physician present at the intake stress test to ensure safety. Finally, while a referring physician may not consider their patient to be an appropriate candidate, many CR programs offer alternative models of care to meet diverse patient needs and are aware of other outpatient resources available with which the physician may not be familiar. Overall, any healthcare provider should have the ability to refer a patient to education at CR based on clinical criteria.

As hypothesized, provision of early outpatient education by CR programs prior to enrollment was shown to increase the likelihood of patients subsequently enrolling in CR. Results are supported by a recently-published manuscript from another Canadian province [27]. Indeed, provision of early outpatient education likely also has ancillary benefits of encouraging earlier adoption of heart-health promoting behaviours, providing reassurance to patients and family members, verifying discharge instructions, and ensuring identification of any clinical issues which may have arisen such as infection. Cardiac patients are often ready to exercise somewhat later than when they need information. Moreover, there can be delays in booking intake exercise stress tests needed to initiate an exercise program, and thus offering an early education session can circumvent any delays this causes.

Moreover, this approach may potentially mitigate any wait time delays in commencing the CR program. Wait time benchmarks have been established by cardiac indication in Canada through clinical consensus [28]. Access delays may reduce enrollment rates because patients may have returned to work, or perceive less need for these services over time following an acute cardiac hospitalization. Of course, the potential impact on CR program capacity warrants further exploration.

Caution is warranted when interpreting these findings, chiefly due to study design. This was a quasi-experimental study. For ethical reasons, cardiac patients could not be randomized to acute care site or ward, nor could we randomize strategy within site due to the potential for contamination. The results herein nevertheless present the pragmatic or real-world effects of strategies to increase CR utilization. There were significant differences in sociodemographic and clinical characteristics of patients by strategy which may have biased results. We controlled for these in subsequent analyses, however a randomized design would be needed to definitively establish the effects of strategy on CR use. In addition, because the referral strategies were not mutually exclusive, interaction between the interventions may have affected the results. Some participants used as the comparison group for a specific strategy may have been exposed to one of the other CR strategies. Overall, there were four (23.5%) inpatient wards offering both strategies. In addition, some participants exposed to EARLY ED were recruited from a ward offering PRE-BOOKED or PRE-APPROVED strategies, possibly influencing the odds ratio for EARLY ED. The authors attempted to mitigate this threat by incorporating all relevant referral strategies in each model. The compelling results of this pragmatic observational study warrant replication in a cluster randomized controlled trial.

The second limitation pertains to measurement. Although self-reported CR referral and enrollment was not verified, there is evidence that supports the ‘almost-perfect’ congruence between self-report and CR site-report data [29]. However, the potential for social desirability biases in participant responses cannot be ruled out. The final limitation pertains to generalizability. The initial response rate and the retention rate suggest some degree of caution in interpreting the findings is warranted. In addition, the present study was conducted in a region where CR services are reimbursed through provincial healthcare coverage, and therefore enrollment rates attained may not be applicable to other regions where patients must pay out-of-pocket for CR.

In conclusion, two readily-implementable strategies were shown to increase CR enrollment, up to 65-84%. This is approximately two to five times greater access than under usual care, suggesting wider adoption of these strategies should be promoted. Randomized controlled trials are needed to confirm the robustness of these strategies in manualized form, as well as comparative effectiveness studies to ascertain the strategy or combination of strategies which can consistently optimize utilization.

## Competing interests

The authors declare that they have no competing interests.

## Authors’ contributions

SG made substantial contributions to conception, design, acquisition and interpretation of data, and drafting the manuscript. KLR undertook data acquisition and analysis as well as drafting the manuscript. RR made substantial contributions to conception and design as well as interpretation of data. PO made substantial contributions to conception and design as well as interpretation of data. SA was involved in acquisition and interpretation of data. MG made substantial contributions to conception and design. SB made substantial contributions to conception and design. DES revised the manuscript critically for important intellectual content and gave final approval of the version to be published. All authors read and approved the final manuscript.
